# Comparative analysis of the association between various serum vitamin D biomarkers and sarcopenia

**DOI:** 10.1002/jcla.23946

**Published:** 2021-08-05

**Authors:** Jun‐Il Yoo, Hye Jin Chung, Bo Gyu Kim, Youn‐Kwan Jung, Kyung‐Wan Baek, Myung‐Geun Song, Min‐Chul Cho

**Affiliations:** ^1^ Department of Orthopaedic Surgery Gyeongsang National University Hospital Jinju Korea; ^2^ College of Pharmacy and Research institute of Pharmaceutical Sciences Gyeongsang National University Jinju Korea; ^3^ Biomedical Research Institute Gyeongsang National University Hospital Jinju Korea; ^4^ Department of Laboratory Medicine Gyeongsang National University Hospital Gyeongsang National University College of Medicine Jinju Korea; ^5^ Institute of Health Science Gyeongsang National University Jinju Korea

**Keywords:** bioavailable 25(OH)D, sarcopenia, vitamin D, vitamin D binding protein

## Abstract

**Background:**

Vitamin D status is associated with muscle strength and maintenance of muscle fibers. However, which serum vitamin D biomarker better reflects sarcopenia remains unclear. The aim of this study was to investigate associations between various serum vitamin D biomarkers (total 25‐hydroxy vitamin D [25(OH)D], bioavailable 25(OH)D, 24,25‐dihydroxyvitamin D [24,25(OH)_2_D], and vitamin D metabolite ratio [VMR]) and sarcopenia.

**Methods:**

The data for 83 hip fracture patients were finally included in the analysis. Sarcopenia was defined according to the Asia Working Group for Sarcopenia (AWGS) criteria. Measurements of 24,25(OH)_2_D and 25(OH)D were made using solid‐phase extraction (SPE) and subsequent liquid chromatography‐tandem mass spectrometry (LC‐MS/MS). Vitamin D binding protein (VDBP) concentration was measured using an enzyme‐linked immunosorbent assay. The VMR was calculated by dividing serum 24,25(OH)_2_D by serum 25(OH)D and then multiplying by 100. Based on total 25(OH)D, VDBP, and albumin concentrations, bioavailable 25(OH)D concentrations were calculated using the equations from the other previous studies.

**Results:**

Bioavailable 25(OH)D levels were significantly (*p* = 0.030) decreased in the sarcopenia group compared with the non‐sarcopenia group. Results of ROC analysis for the diagnosis of sarcopenia using serum level of bioavailable of 25(OH)D revealed that the cutoff point for bioavailable 25(OH)D was 1.70 ng/ml (AUC = 0.649, *p* < 0.001). In the group with a bioavailable 25(OH)D less than 1.70 ng/ml, the incidence of sarcopenia increased by 3.3 times (odds ratio: 3.33, *p* = 0.013).

**Conclusion:**

We demonstrated that bioavailable 25(OH)D was associated with sarcopenia among the various serum vitamin D biomarkers. Bioavailable vitamin D might be helpful for assessing the risk of sarcopenia.

## Graphical abstract

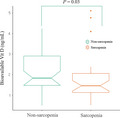

## 1 INTRODUCTION

Vitamin D is mainly synthesized in the skin of people exposed to sunlight, and some can also be obtained from the diet. Vitamin D needs a two‐step hydroxylation (25‐hydroxylation and 1α‐hydroxylation) to be in its biologically active form. The first hydroxylation, 25‐hydroxylation, occurs in the liver. The product, 25‐hydroxy vitamin D [25(OH)D], is transported to the kidneys bound to vitamin D binding protein (VDBP). It is converted to 1α, 25‐dihydroxyvitamin D (1α, 25(OH)_2_D), an active form of vitamin D in the kidney.^1^, ^2^

It is well known that vitamin D primarily plays a critical role in the regulation of calcium homeostasis. Skeletal muscles may require vitamin D and calcium for normal development and maintenance of function. It was previously reported that vitamin D deficiency and inactivating mutations in vitamin D receptor (VDR) are associated with muscle weakness in humans and mouse models.^3^, ^4^, ^5^

Sarcopenia is an age‐related clinical condition characterized by a gradual and generalized loss of skeletal muscle mass with a decrease in strength and physical capacity.^6^ It is considered to be one of the risk factors for adverse events in older people, including delirium, disability, institutionalization, and even death.^7^, ^8^ Nowadays, there is an increasing trend in the prevalence of sarcopenia, which is probably related to an increase in human life expectancy.^9^

Vitamin D deficiency is common among older people around the world. Older people are particularly prone to the development of vitamin D insufficiency or deficiency for following reasons: a reduced cutaneous synthesis in the skin, decreased daily sun exposure, and chronic diseases of organs related to vitamin D metabolism.^9^, ^10^, ^11^ Many prospective studies have examined the role of vitamin D in muscle strength and physical performance of older adults.^12^, ^13^, ^14^ Roles of vitamin D and VDR in muscles have been well described in numerous studies and reviews.^4^, ^15^, ^16^

Several vitamin D biomarkers have been suggested for evaluating vitamin D status in the body. Commonly, vitamin D status is assessed by one measurement of serum 25(OH)D concentration. Generally used criteria for the evaluation of vitamin D status are as follows: vitamin D deficiency, <20 ng/ml; vitamin D insufficiency, 20–30 ng/ml; and vitamin D sufficiency, >30 ng/ml.^2^, ^17^, ^18^ However, some recent studies have suggested that 25(OH)D alone may not reflect accurate vitamin D status.^19^, ^20^, ^21^, ^22^ As alternative indicators for assessing vitamin D status, bioavailable 25(OH)D, serum 24,25‐dihydroxyvitamin D (24,25(OH)_2_D), and the ratio of serum 24,25(OH)_2_D to 25(OH)D known as vitamin D metabolite ratio (VMR) have been proposed.

Bioavailable 25(OH)D is a free or albumin‐bound form of 25(OH)D that is not bound to VDBP. Its concentration is affected by serum VDBP, albumin concentration, and also VDBP encoding *GC* gene genotype. VDBP concentration can be altered under various conditions. VDBP is increased under hyper‐estrogen state such as pregnancy, whereas it is decreased in certain disease states including severe hepatic disease.^23^, ^24^, ^25^, ^26^ The *GC* gene encodes VDBP and two single nucleotide polymorphisms (SNPs), rs7041 and rs4588, generating three major polymorphic isoforms of VDBP: Gc1f, Gc1s, and Gc2.^27^, ^28^ Since the affinity of VDBP for vitamin D is isoform‐dependent, the *GC* genotype plays an important role in determining serum bioavailable 25(OH)D levels.^25^, ^26^, ^28^

24,25(OH)_2_D is the major product of catabolism of 25(OH)D. Because enzymatic synthesis of 24,25(OH)_2_D is directly proportional to the concentration of 25(OH)D substrate, concentrations of both metabolites in circulation are strongly correlated.^29^ Furthermore, expression of 24‐hydroxylase enzyme (CYP24A1) that converts 25(OH)D to 24,25(OH)_2_D is regulated in part by vitamin D receptor activity.^30^, ^31^ Since the production of 24,25(OH)_2_D is regulated by the concentration of 25(OH)D and feedback through the vitamin D receptor, concentration of 24,25(OH)_2_D might be a better indicator of vitamin D status than 25(OH)D itself.^32^ Recent findings have also suggested that the adequacy of vitamin D may be reflected by VMR.^22^, ^33^ This ratio also depend primarily on CYP24A1 expression, which is downregulated in vitamin D deficiency. Therefore, VMR could also be an alternative indicator that accurately reflects vitamin D status.

It is well known that vitamin D status is associated with muscle strength and maintenance of muscle fibers. However, which serum vitamin D biomarker better reflects sarcopenia remains unclear. Therefore, the objective of the present study was to investigate the relationship between various vitamin D biomarkers including 25(OH)D, bioavailable vitamin D, 24,25(OH)_2_D, and VMR through patients with sarcopenia control study in order to elucidate which biomarkers may better reflect sarcopenia.

## 2 MATERIALS AND METHODS

### 2.1 Study subjects

**FIGURE 1:**
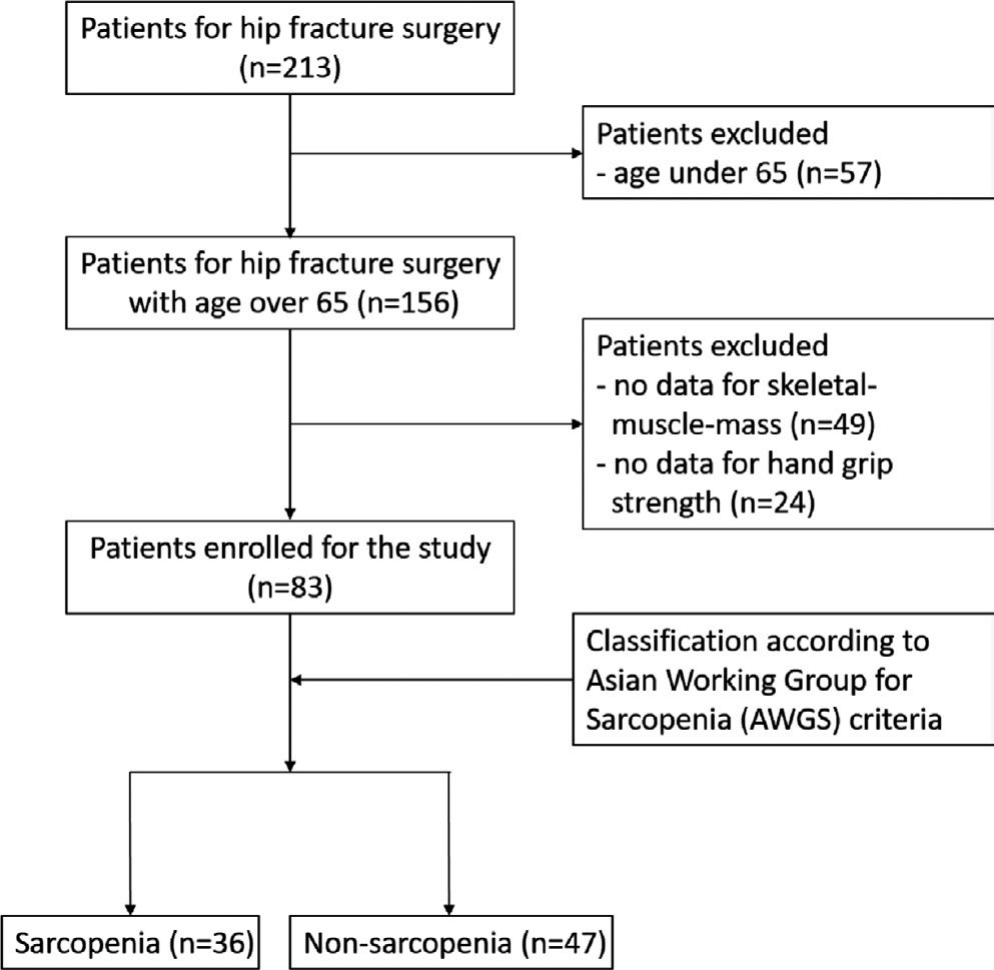
Flow diagram of patients involved in the study

Data of 213 patients after a hip fracture (HF) surgery from May 2018 to December 2019 were collected. To diagnose sarcopenia according to Asian Working Group for Sarcopenia (AWGS) criteria, patients younger than 65 years (*n* = 57) and the patients who had no data for skeletal muscle mass (*n* = 49) or hand grip strength (*n* = 24) were excluded. After these exclusions, a total of 83 HF patients (sarcopenia, *n* = 36; non‐sarcopenia, *n* = 47) were finally included in the analysis. The study design is displayed in the flow diagram in Figure 1.

Demographic and laboratory data including age, sex, height, weight, serum albumin, calcium, parathyroid hormone, alkaline phosphatase, aspartate aminotransferase, and alanine aminotransferase were collected from electronic medical records. At the time of study enrollment, blood samples including serum and whole blood were collected. Serum and leukocytes were then separated and stored at −80°C deep freezer until analysis. The study protocol was approved by the Institutional Review Board (IRB) of Gyeongsang National University Hospital (IRB number: 2017‐12‐004). Written informed consent was obtained from all the participants.

### 2.2 Diagnosis of sarcopenia

Body composition was measured using a whole‐body dual X‐ray absorptiometry (DEXA), for which a QDR 4500A apparatus (Hologic) was employed. Bone mineral content, fat mass, and lean soft tissue mass were measured separately for each part of the body, including arms and legs. Lean soft tissue masses of arms and legs were almost equal to skeletal muscle mass. Absolute muscle mass is known to correlate with height. Thus, skeletal muscle mass index (SMI) was calculated with the following formula: lean mass (kg)/height^2^ (m^2^), which was directly analogous to body mass index (BMI: weight [kg]/height^2^[m^2^]). Arm SMI was defined as (arm lean mass [kg]/height^2^[m^2^]). Leg SMI was defined as (leg lean mass [kg]/height^2^ [m^2^]). Appendicular SMI was defined as the sum of arm and leg SMIs.

Muscle strength was assessed by handgrip strength using an analogue dynamometer (TK 5001 Grip‐A; Takei). In a sitting position, grip with maximal strength was measured when the elbow was flexed at 90 degrees with the shoulder attached to the torso and the wrist maintaining a neutral posture (0 degrees). Sarcopenia was defined according to the AWGS criteria for low muscle strength (hand grip strength below 18 kg in women and 28 kg in men) and low muscle mass (SMI below 5.4 kg/m^2^ in women and 7.0 kg/m^2^ in men).

### 2.3 Vitamin D measurements

Measurements of 24,25(OH)_2_D and 25(OH)D were made using solid‐phase extraction (SPE) and subsequent liquid chromatography‐tandem mass spectrometry (LC‐MS/MS), as described by van den Ouweland et al.^34^ with slight modifications. Following the addition of the internal standard, stable isotope‐labeled d_6_‐24,25(OH)_2_D and d_6_‐25(OH)D to 200 μl of serum sample, methanol was added, vortex‐mixed, and then kept for 10 min at 4°C for protein precipitation. After centrifugation at 4°C at 12,000 *g* for 10 min, the supernatant was mixed with phosphate‐buffered saline and loaded onto an SPE cartridge. After performing SPE, the elution fraction was evaporated under a vacuum. The dried residue was reconstituted in 75% methanol, and then, 5 μL was injected into the LC‐MS/MS system for analysis. The LC‐MS/MS system consisted of an Agilent 1260 HPLC system (Agilent Technologies) with an Agilent 6460 triple quadrupole mass spectrometer (Agilent Technologies) equipped with an electrospray ionization (ESI) source. Kinetex^®^ Biphenyl column (2.6 μm, 3.0 × 100 mm; Phenomenex) and Poroshell^®^ 120 EC‐C 18 column (2.7 μm, 3.0 × 50 mm; Agilent Technologies) were used for HPLC separation of 24,25(OH)_2_D and 25(OH)D, respectively. The HPLC mobile phase consisted of 0.1% aqueous formic acid and methanol, and a gradient program was used at a flow rate of 0.4 ml/min. The multiple reaction monitoring (MRM) detection method was used for the detection of analytes. Transitions monitored were *m*/*z* 417 → 381 for 24,25(OH)_2_D, *m*/*z* 423 → 387 for d_6_‐24,25(OH)_2_D, *m*/*z* 401 → 383 for 25(OH)D, and *m*/*z* 407 → 389 for d_6_‐25(OH)D. The limits of quantitation of 24,25(OH)_2_D and 25(OH)D were 0.2 and 2 ng/ml, respectively.

### 2.4 VDBP assay and *GC* genotyping

Vitamin D binding protein concentration was measured using an enzyme‐linked immunosorbent assay (ELISA) kit (R&D Systems) according to the manufacturer's protocol.

For *GC* gene genotyping, genomic DNA was isolated from peripheral blood leukocytes using a DNeasy Blood and Tissue Kit (Qiagen) according to the manufacturer's instructions. *GC* genotyping for rs7041 (c.1296T > G; p. Asp432Glu) and rs4588 (c.1307C > A; p. Thr436Lys) was performed using a TaqMan SNP Genotyping Assay (Thermo Fisher Scientific) and an ABI ViiA 7 Real‐Time PCR System (Applied Biosystems) according to each manufacturer's instructions and described in the previous study.^35^

### 2.5 Calculation of VMR and bioavailable 25(OH)D concentration

Vitamin D metabolite ratio was calculated by dividing serum 24,25(OH)_2_D by serum 25(OH)D and then multiplying by 100.^32^ Bioavailable 25(OH)D concentrations were calculated using the equations reported in previous studies with serum 25(OH)D, VDBP, albumin concentrations, and *GC* genotype.^35^, ^36^

### 2.6 Statistical analysis

The sample size was calculated to be 66 considering an expected sensitivity of 90%, an expected specificity of 50%, a disease prevalence of 35%, an acceptable precision of 15%, and a significance level of 0.05. Finally, we decided to recruit more than 83 subjects, considering a dropout rate of 20%.^37^

To compare means and proportions of each group, Student's *t* test and chi‐squared (χ2) test were employed. The Pearson correlation test was used for correlation analysis. A receiver operating characteristic (ROC) curve analysis was also performed to identify the cutoff value for diagnosis of sarcopenia using bioavailable 25(OH)D. All statistical tests were two‐tailed. Statistical significance was defined at *p* < 0.05. All statistical calculations were performed using SPSS Statistics V.22 (SPSS Inc.) and software R (v 3.1.0; The R 100 Foundation).

## 3 RESULTS

### 3.1 Demographic characteristics and laboratory test results

A total of 83 patients were enrolled (35 in the sarcopenia group and 48 in the non‐sarcopenia group). The age of patients was 74.1 ± 12.3 years in the sarcopenia group and 70.7 ± 10.0 years in the non‐sarcopenia group. The male‐to‐female ratio was 0.59 in the sarcopenia group and 0.33 in the non‐sarcopenia group. Among demographic characteristics, age, sex, height, and weight were not significantly different between the two groups (sarcopenia vs. non‐sarcopenia). Among laboratory test results, serum levels of albumin, calcium, alkaline phosphatase, aspartate aminotransferase (AST), and alanine aminotransferase (AST) were not significantly different either between the two groups (sarcopenia vs. non‐sarcopenia). However, parathyroid hormone (PTH) was significantly higher in the non‐sarcopenia group than in the sarcopenia group (*p* = 0.015). Demographic characteristics and laboratory test results of patients are shown in Table 1.

**TABLE 1:** Patient demographics and laboratory test results for the two study groups

	Sarcopenia (*n* = 35)	Non‐sarcopenia (*n* = 48)	*p* Value
Age (years)	74.1 ± 12.3	70.7 ± 10.0	0.169
Sex			
Male	13 (37.1%)	12 (25.0%)	0.234
Female	22 (62.9%)	36 (75.0%)	
Height (cm)	154.6 ± 21.7	155.6 ± 10.1	0.770
Weight (kg)	53.6 ± 8.3	56.7 ± 9.9	0.142
BMI (kg/cm^2^)	23.1 ± 3.3	27.1 ± 31.9	0.082
Albumin *(*g/dl)	4.0 ± 0.5	4.2 ± 0.5	0.111
PTH (pg/ml)	33.9 ± 44.3	44.3 ± 20.6	0.015
Calcium (mg/dl)	7.1 ± 7.9	7.9 ± 3.5	0.343
ALP (U/L*)*	105.8 ± 42.2	104.4 ± 49.9	0.894
AST (U/L*)*	21.7 ± 8.1	21.7 ± 8.1	0.995
ALT (U/L*)*	14.4 ± 6.8	15.6 ± 7.5	0.480

Note

Data were presented as mean ± standard deviation or number (percentage).

Abbreviations: ALP, alkaline phosphatase; ALT, alanine aminotransferase; AST, aspartate aminotransferase; BMI, body mass index; PTH, parathyroid hormone.

### 3.2 Comparison of serum vitamin D biomarkers by the presence of sarcopenia

Bioavailable 25(OH)D levels were significantly (*p* = 0.030) decreased in the sarcopenia group than in the non‐sarcopenia group (Table 2). Levels of 24,25(OH)_2_D were decreased with marginally significance (*p* = 0.087) in the sarcopenia group than in the non‐sarcopenia group. Total 25(OH)D, VDBP, and VMR were not significantly different between the two groups (sarcopenia vs. non‐sarcopenia) (Table 2).

**TABLE 2:** Level of serum vitamin D biomarkers by presence of sarcopenia

	Sarcopenia (*n* = 35)	Non‐sarcopenia (*n* = 48)	*p* Value
Total 25(OH)D (ng/ml)	19.21 ± 10.82	22.31 ± 10.15	0.185
VDBP (µg/ml)	258.22 ± 84.66	281.64 ± 90.84	0.236
Bioavailable 25(OH)D (ng/ml)	1.70 ± 0.87	2.18 ± 1.06	0.030
24,25(OH)_2_D (ng/ml)	0.91 ± 0.73	1.21 ± 0.77	0.087
VMR	4.21 ± 2.04	4.36 ± 2.03	0.741

Note

Data were presented as mean ± standard deviation.

Abbreviations: 24,25(OH)_2_D, 24,25‐dihydroxyvitamin D; 25(OH)D, 25‐hydroxy vitamin D; VDBP, vitamin D binding protein; VMR, vitamin D metabolite ratio.

### 3.3 Vitamin D status according to total 25(OH)D

**FIGURE 2:**
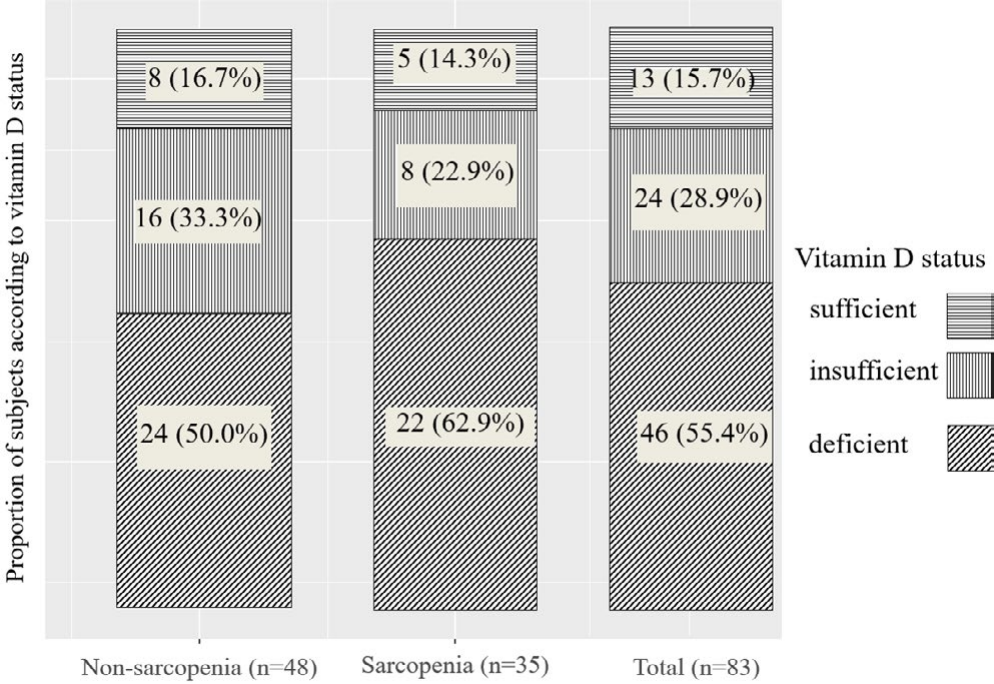
Proportion of subjects according to vitamin D status based on total 25(OH)D concentration; sufficient (≥30 ng/ml); insufficient (20–29 ng/ml); and deficient (≤20 ng/ml). 25(OH)D, 25‐hydroxy vitamin D

Based on total 25(OH)D level, the status of vitamin D by deficiency, insufficiency, and sufficiency was evaluated in total, non‐sarcopenia, and sarcopenia groups (Figure 2). In total patients, numbers of patients with vitamin D deficiency, insufficiency, and sufficiency were 46 (55.4%), 24 (28.9%), and 13 (15.7%), respectively. In the sarcopenia group, numbers of patients with vitamin D deficiency, insufficiency, and sufficiency were 22 (62.9%), 8 (22.9%), and 5 (14.3%), respectively. In the non‐sarcopenia group, numbers of patients with vitamin D deficiency, insufficiency, and sufficiency were 24 (50.0%), 16 (33.3%), and 8 (16.7%), respectively. The ratio of vitamin status was not significantly (*p* = 0.485) different between the two groups.

### 3.4 Correlation analysis of variables associated with sarcopenia

**FIGURE 3:**
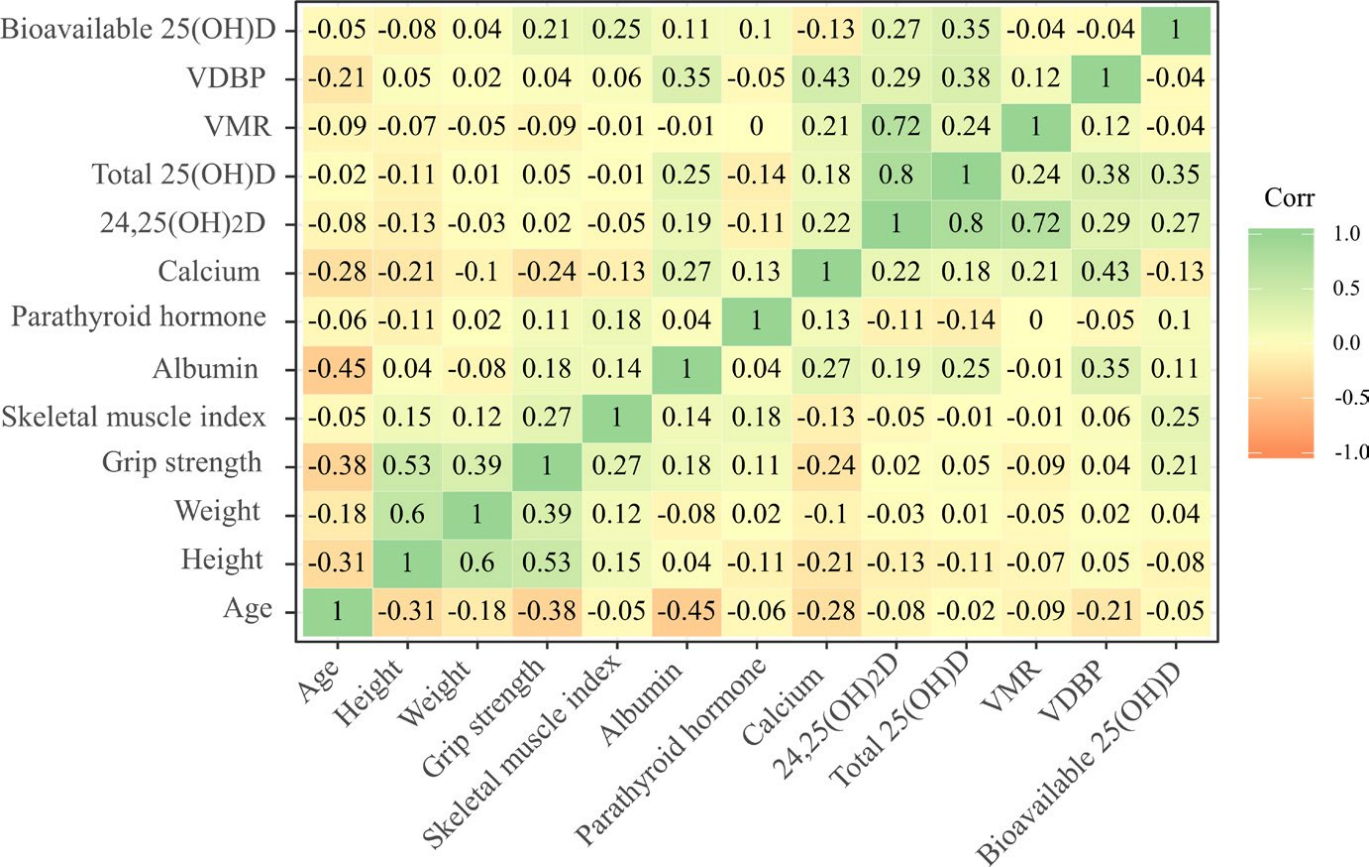
Correlogram of variables associated with sarcopenia. 24,25(OH)_2_D, 24,25‐dihydroxyvitamin D; 25(OH)D, 25‐hydroxy vitamin D; VDBP, vitamin D binding protein; VMR, vitamin D metabolite ratio

Correlation analysis was performed with indicators related to sarcopenia and various vitamin D biomarkers. Results are shown in Figure 3. There were no statistically significant correlations between indicators related to sarcopenia and vitamin D biomarkers.

### 3.5 Receiver operating characteristic curve analysis for the diagnosis of sarcopenia using bioavailable 25(OH)D

**FIGURE 4:**
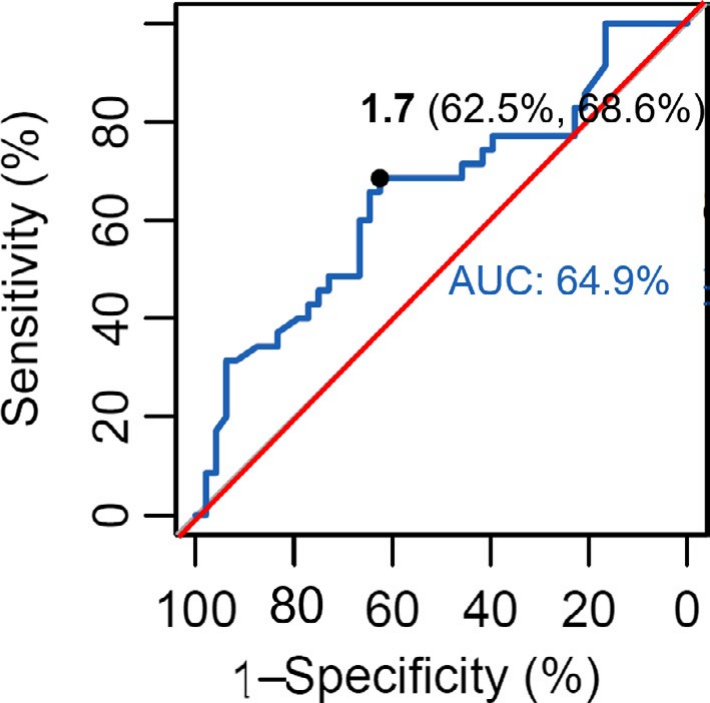
ROC curve analysis for the diagnosis of sarcopenia using bioavailable 25(OH)D. 25(OH)D, 25‐hydroxy vitamin D; ROC, receiver operating characteristic

Receiver operating characteristic curve analysis was performed for the diagnosis of sarcopenia using serum levels of bioavailable of 25(OH)D. Results of ROC curve analysis showed that the cutoff point for bioavailable 25(OH)D was 1.70 ng/ml (AUC = 0.649, *p* < 0.001) (Figure 4). ROC curve analysis produced an AUC of 0.649 (95% confidence interval: 0.23–0.47; *p* = 0.021). The cutoff value of bioavailable 25(OH)D for maximum sensitivity (62.50%) and specificity (68.60%) was 1.70 ng/ml (Figure 4). In the group with a bioavailable 25(OH)D less than 1.70 ng/ml, the incidence of sarcopenia increased by 3.3 times (odds ratio: 3.33; 95% confidence interval: 1.32–8.39; *p* = 0.013).

### 3.6 Genotype and allele frequencies of *GC* gene encoding VDBP

In total study subjects, Gc1f‐1f (*n* = 25, 30.1%) was the most genotype, followed by Gc1s‐1f genotype (*n* = 22, 26.5%) and Gc2‐1f genotype (*n* = 19, 22.9%). The three most frequent genotypes were in the order of Gc1s‐1f > Gc1f‐1f > Gc2‐1f in the sarcopenia group and Gc1f‐1f > Gc2‐1f > Gc1s‐1f in non‐sarcopenia group. There was no significant difference in genotype distribution and frequency between the two groups (*p* = 0.532; Table 3). In total study patients, Gc1f (*n* = 91, 54.8%) was the most common allele, followed by Gc1s (*n* = 40, 24.1%) and Gc2 (*n* = 35, 21.1%) The three most frequent alleles were in the order of Gc1f > G1s > Gc2 in both sarcopenia and non‐sarcopenia groups. There was no significant difference in allele distribution between the two groups (*p* = 0.785; Table 3).

**TABLE 3:** *GC* genotype and allele frequencies

	All patients (*n* = 83)	Sarcopenia (*n* = 35)	Non‐sarcopenia (*n* = 48)	*p* Value
*GC* genotype				
Gc1f‐1f	25 (30.1%)	11 (31.4%)	14 (29.2%)	0.532
Gc1s‐1f	22 (26.5%)	12 (34.3%)	10 (20.8%)	
Gc1s‐1s	4 (4.8%)	1 (2.9%)	3 (6.3%)	
Gc2‐1f	19 (22.9%)	6 (17.1%)	13 (27.1%)	
Gc2‐1s	10 (12.1%)	3 (8.6%)	7 (14.6%)	
Gc2‐2	3 (3.6%)	2 (5.7%)	1 (2.1%)	
*GC* allele				
Gc1f	91 (54.8%)	40 (57.1%)	51 (53.1%)	0.785
Gc1s	40 (24.1%)	17 (24.3%)	23 (24.0%)	
Gc2	35 (21.1%)	13 (18.6%)	22 (22.9%)	

Note

Data were presented as number (percentage).

## 4 DISCUSSION

In addition to the currently commonly used total 25(OH)D, various other biomarkers have been suggested as an indicator for evaluating vitamin D status. The newly proposed vitamin D biomarkers include bioavailable vitamin D, 24,25(OH)_2_D, and VMR.^25^, ^29^ Many previous studies have evaluated serum vitamin D levels with sarcopenia. However, these studies analyzed correlations of serum total 25(OH)D with sarcopenia.^38^, ^39^, ^40^ In this study, associations between various serum vitamin D biomarkers, including 25(OH)D, bioavailable vitamin D, 24,25(OH)_2_D, and VMR, and sarcopenia were analyzed. Results demonstrated that bioavailable vitamin D was associated with sarcopenia and could be the best biomarker reflecting sarcopenia among all serum vitamin D biomarkers tested. To the best of our knowledge, the present study is the first research to analyze various serum biomarkers simultaneously in sarcopenia patients.

In this study, the enrolled patient group was classified into two groups, sarcopenia and non‐sarcopenia, and various vitamin D biomarkers were compared in the two groups. Total 25(OH)D levels showed no significant difference between sarcopenia and non‐sarcopenia groups. However, bioavailable 25(OH)D level was significantly lower in the sarcopenia group. The bioavailable vitamin D is either free or weakly bound to albumin, not bound to the vitamin D transporter, VDBP. Therefore, bioavailable vitamin D is considered to be a vitamin D that exhibits an immediate response of vitamin D because it is relatively easier to move into the nucleus of target cells by binding to vitamin D receptors than in the form combined with VDBP.^23^, ^24^ This mechanism of action was asserted by the “free hormone hypothesis” suggested by the mechanism of action of other steroid hormones having a steroid structure such as vitamin D. According to this hypothesis, most of the steroid hormones are bound to the binding proteins that act as transporters in the blood; thus, they do not exhibit biological activity, and free hormones that do not bind to the binding proteins represent biological importance.^41^, ^42^ Results of the present study may support this free hormone hypothesis about vitamin D. In addition, our findings suggest that it would be more appropriate to evaluate the vitamin D status in patients with suspected sarcopenia, not as bioavailable vitamin D, rather than total 25(OH)D.

Considering results of previous studies on the role of vitamin D in the differentiation and maintenance of muscle fibers,^43^, ^44^ it could be assumed that low concentration of bioavailable vitamin D may influence the onset of sarcopenia. Therefore, concentration of bioavailable vitamin D could be used to assess the risk of sarcopenia. Studies on the usefulness of bioavailable 25(OH)D in many different diseases are actively underway; however, the reference range for using bioavailable 25(OH)D as a clinical indicator or the cutoff value for diagnosis of specific diseases has not been established. In the present study, we suggested bioavailable vitamin D concentration of 1.70 ng/ml as a cutoff value for assessing the risk of developing sarcopenia. In fact, in our study, when odds ratio analysis was performed based on bioavailable 25(OH)D concentration, it was confirmed that the relative risk for sarcopenia increased by 3.3 times compared with 1.70 ng/ml or more for less than 1.70 ng/ml. However, in ROC curve analysis with bioavailable 25(OH)D 1.70 ng/ml as cutoff for the diagnosis of sarcopenia, AUC was only 0.649. Therefore, bioavailable 25(OH)D alone has limitations in diagnosing sarcopenia. However, it implies that it could be used as an auxiliary criterion to predict the risk of sarcopenia. However, it is difficult to draw a conclusive conclusion that is clinically useful based on findings of this study alone. More well‐designed and large‐scale studies will be required.

In the present study, VDBP concentration was 271.74 ± 88.52 µg/ml (mean ± SD) for all subjects enrolled, which was significantly (*p* < 0.0001) higher than that in a previously reported VDBP concentration of 166.47 ± 36.36 µg/ml in healthy people.^35^ VDBP is an acute‐phase reactant. Its concentration is known to increase after trauma due to increases in cytokine and glucocorticoid.^45^ Therefore, it was thought that VDBP level was increased in patients with hip fractures as participants of our study than that in the healthy control group. In fact, in our study, serum VDBP levels did not show statistically significant differences between sarcopenia and non‐sarcopenia groups, although bioavailable 25(OH)D levels were different between the two groups. It was known that factors affecting bioavailable 25(OH)D level were serum total 25(OH)D, albumin, VDBP level, and VDBP genotype.^35^, ^36^ However, in the present study, these factors were not significantly different between the two groups (sarcopenia vs. non‐sarcopenia). Other factors not yet known might have influenced bioavailable 25(OH)D level. Therefore, further research is needed to clarify finding of the present study.

Vitamin D deficiency is common in the older population all over the world. Older patients are particularly vulnerable to the development of vitamin D insufficiency or deficiency for some causes such as a reduced cutaneous synthesis, decreased daily sun exposure, and/or various diseases such as chronic renal failure and gastrointestinal malabsorption.^10^ In this study, the elderly with an average age of 72.1 years showed a high rate of vitamin D deficiency or deficiency of 84.3% in all patients. In the sarcopenia group, the rate of subjects with vitamin D deficiency was higher than that of the non‐sarcopenia group, although the difference between the two was not statistically significant (82.8% vs. 83.3%, *p* = 0.173).

Major *GC* genotype and allele frequencies are known to vary among ethnicities.^28^ *GC* allele frequencies in Koreans have been reported in 203 patients with chronic obstructive pulmonary disease and 157 control subjects.^46^ In the present study, Gc1f‐Gc1f (30.1%) was the most frequent genotype, followed by Gc1s‐Gc1f (26.5%), Gc2‐Gc1f (22.9%), and Gc2‐ Gc1s (12.1%), consistent with frequencies observed in the previous study.^46^ *GC* genotype and allele frequency failed to have any significant correlation with the presence or absence of sarcopenia.

The association between vitamin D and sarcopenia has been explored in various ways in many studies.^4^, ^47^, ^48^, ^49^, ^50^ The mechanism of action of vitamin D on target cells, including skeletal muscle cells, can be divided into two stages.^51^ The first stage is the stage until vitamin D present in the general circulation binds to VDR of the target cell, and the second stage is the downstream action stage that occurs after binding to the VDR. The present study analyzed the association between serum vitamin D levels and sarcopenia, the first of the two stages above. Research on the second stage is also being actively performed. Studies have shown that VDR levels in skeletal muscle of older adults decrease with age and are lower than those of younger peoples.^47^, ^48^ Furthermore, it has also been reported that the polymorphism of VDR gene is associated with sarcopenia.^48^, ^52^ To further clarify the relationship between vitamin D and sarcopenia, both steps mentioned above should be considered due to the mechanism of action of vitamin D on skeletal muscle cells.

The present study has three main limitations. First, since all enrolled study subjects were patients with hip fractures, the influence of vitamin D–related factors by fracture could not be excluded. Therefore, subjects may not represent general sarcopenia patients. Second, environmental factors that could affect vitamin D concentration, including food, outdoor activity period, use of sunscreen, and vitamin D supplement intake, were not surveyed. Third, our study enrolled a total of 83 patients, including 35 sarcopenia patients, with a relatively small number of subjects. Because of the small sample size, it might not be possible to clarify the association between VDBP polymorphic isoform and sarcopenia. Due to these limitations, it was impossible to eliminate all confounding factors in the analysis of various vitamin D status biomarkers. Despite these limitations, this was the first report showing that bioavailable 25(OH)D was associated with sarcopenia. It could better reflect sarcopenia than other vitamin D biomarkers.

In summary, among various serum vitamin D biomarkers, bioavailable 25(OH)D was associated with sarcopenia. Thus, bioavailable vitamin D might be used as an auxiliary marker for assessing the risk of sarcopenia.

## CONFLICT OF INTEREST

The authors declare that this research is not related to any commercial or financial interests.

## AUTHOR CONTRIBUTIONS

JIY and MCC contributed to the study planning. HJC, BGK, and YKJ performed the experiments. JIY, KWB, and MGS contributed to the inclusion of patients. JIY and MCC contributed to analysis and interpretation of the data. All authors contributed to writing the paper and approved the final version for publication.

## Data Availability

The data that support the findings of this study are available from the corresponding author upon reasonable request.
